# *In Vitro* Properties of Orthodontic Adhesives with Fluoride or Amorphous Calcium Phosphate

**DOI:** 10.1155/2011/583521

**Published:** 2011-09-06

**Authors:** Clara Ka Wai Chow, Christine D. Wu, Carla A. Evans

**Affiliations:** ^1^Markham Orthodontic Specialty, 3000 Highway 7 E Suite 208, Markham, ON, Canada L3R 6E1; ^2^University of Illinois at Chicago, 801 S. Paulina Street MC841, Chicago, IL 60612-7211, USA

## Abstract

This *in vitro* study evaluated the efficacy of orthodontic adhesives with fluoride or amorphous calcium phosphate (ACP) in reducing bacterial adhesion and enamel demineralization. Forty human premolars each sectioned buccolingually into three parts were bracketed with control resin (Transbond XT) or adhesives containing ACP (Aegis Ortho) or fluoride (QuickCure). Artificial lesions induced by pH cycling were examined by X-ray photoelectron spectrophotometry (XPS) and polarized light microscopy (PLM). After 28 days, Aegis Ortho demonstrated the lowest calcium and phosphorous content by XPS analysis. After 42 days, reductions in lesion depth areas were 23.6% for Quick Cure and 20.3% for Aegis Ortho (*P* < 0.05). In the presence of 1% sucrose, adhesion of *Streptococcus mutans* to Aegis Ortho and Quick Cure was reduced by 41.8% and 37.7% (*P* < 0.05) as compared to Transbond XT. Composites containing ACP or fluoride reduced bacterial adherence and lesion formation as compared to a composite without ACP or fluoride.

## 1. Introduction

Decalcification around orthodontic brackets is a common risk of fixed orthodontic treatment. The incidence of decalcification has been reported between 2–96% [1, 2]. White spot lesions have been attributed to prolonged accumulation and retention of bacterial plaque and could be seen within 4 weeks of treatment without fluoride application [2, 3]. These subsurface lesions can be remineralized in a plaque-free environment provided that the surface layer is still intact [4]. *Streptococcus mutans* is a known caries initiator that has been isolated from bacterial colonies developed in contact with used brackets [5]. Initial colonization of *S. mutans* to orthodontic bonding materials occurred after three days [6]. Hence, there is a need for a bonding material that combats microbial attack to prevent enamel decalcification [5]. 

Incorporating preventive agents in orthodontic bonding composite is a potential method to reduce white spot lesions during orthodontic treatment, but it was found that some efforts impaired the physical properties or caused a rapid decrease of antibacterial effect [1, 7]. Even when combined adhesives have desired properties, commercialization would be difficult [8, 9] because approval from the Food and Drug Administration (FDA) requires classification of these agents as a drug. However, the FDA has approved the use of amorphous calcium phosphate (ACP) in products such as Recaldent (Recaldent Pty. Ltd., Melbourne, Australia), a paste that contains casein phosphopeptide-amorphous calcium phosphate (CPP-ACP).

Under normal conditions, there is only sufficient calcium and phosphate in the saliva to maintain the equilibrium or repair the tooth very slowly. ACP is a biocompatible intermediate in hydroxyapatite (HAP) formation. It provides a sustained release of calcium and phosphate ions when the pH drops below 5.8 [10, 11]. The overall mechanical properties of the ACP composites are inferior to conventional dental composites [12]. Using microradiography, it was reported that artificially produced caries-like lesions in bovine teeth coated with ACP-filled composites recovered 71% ± 33% of their lost mineral content [13]. ACP has been applied to dental uses in topical and bleaching gels, toothpastes, mouthrinses, and sugar-free gum; new delivery systems continue to be proposed [14]. Aegis Ortho is a commercialized orthodontic composite with ACP.

This study addresses the need to reduce iatrogenic caries during orthodontic treatment and thereby improve the effectiveness of care. The goals of this study are as follows.

1. To investigate the* in vitro* adherence of *S. mutans *to three test composites, including one containing amorphous calcium phosphate (ACP).
2. To evaluate the effect of three test composite materials on* in vitro* demineralization of human enamel using polarized light microscopy (PLM) and X-ray photoelectron spectrophotometry surface analysis (XPS).

## 2. Materials and Methods

**Figure 1:**
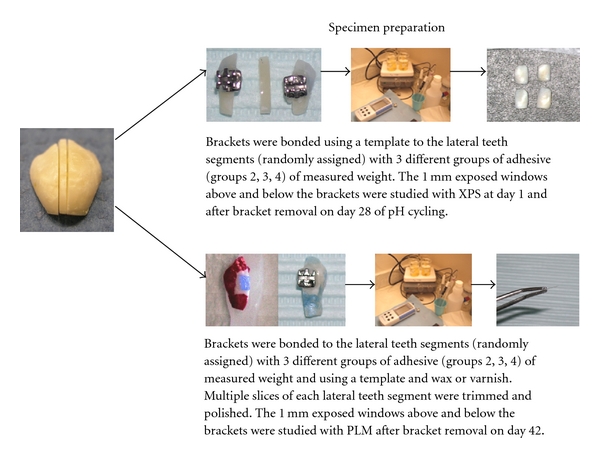
Details of specimen preparation.

Forty extracted noncarious large human premolars were obtained and stored in 0.1% thymol at 4°C until use. Subsequently the remaining soft tissue, calculus, and bone were removed from the teeth with a dental scaler or razor blade. The root portions were removed from the crowns by separating disks. The tooth crowns had fluoride-free prophylaxis and were rinsed in deionized water and air dried. Ten teeth were assigned to the XPS experiment and 30 to the PLM experiment, including the teeth used in pilot PLM studies. Each crown was sectioned buccolingually into three parts as shown in Figure 1 using a diamond wafering blade (Buehler Ltd., Evanston, IL). For sectioning the teeth into three segments, each tooth was first fixed in epoxy formed in a standard cylinder. Then the specimen was sectioned with extreme caution with a diamond blade. For the PLM slices, the specimens were oriented in red wax in cylinders to facilitate finer trimming and polishing. For each tooth, the two lateral segments, each about 3 mm, were wider than the central segment to accommodate placement of orthodontic brackets. The 1-2 mm wide central segments were reserved as nonbracketed, non, adhesive, and not pH-cycled control specimens of enamel. The samples were assigned to control and treatment groups using a random number table. This project was classified as exempt research that does not involve human subjects as defined by the university Office for the Protection of Human Subjects. 

The three adhesives tested were as follows.

1. Transbond XT (3M Unitek, Monrovia, CA), a light cure composite without fluoride or ACP. It has been used as a standard in bonding studies and has been compared in this study as a composite resin control.
2. Quick Cure (Reliance Orthodontic Products, Itasca, IL), a light cure composite with fluoride.
3. Aegis Ortho (Bosworth Co., Skokie, IL), a light cure composite with 38% ACP fillers.

### 2.1. The Brackets

A template was used to position Synergy bicuspid brackets (Rocky Mountain Orthodontics, Denver, CO) on the lateral tooth segments. For the PLM experiment, acid-resistant varnish (nail polish) was painted to within 1 mm area occlusal and apical to each bracket, leaving exposed windows above and below the bracket. This step was not performed in the XPS portion of the study. The bracket area was etched with 37% phosphoric acid gel. Each premolar bracket was bonded with the designated orthodontic adhesive based on the study group, according to the orthodontic adhesive manufacturer's directions. All flash was removed and light cured at 450 nm (Pac-Dent LED Light, Rocky Mountain Orthodontics, Denver, CO).

### 2.2. *In Vitro * pH Cycling

The tooth sections were placed in artificial saliva solution for 12 hours before subjecting them to a demineralizing solution. The artificial saliva solution consisted of 20 mmol/L NaHCO_3_, 3 mmol/L NaH_2_PO_4_, and 1 mmol/L CaCl_2_ at neutral pH. The demineralizing solution consisted of 2.2 mmol/L Ca^2+^, 2.2 mmol/L PO_4_^3−^, 50 mmol/L acetic acid at pH 4.4. All solutions were made with deionized water and measured using a pH/mV meter (Accumet Portable, Fisher Scientific, Pittsburgh, PA) and calcium electrode (Thermo Electron Co., Beverly, MA). The tooth sections were cycled between artificial saliva and demineralizing solutions and exposed to the demineralizing solution for one-hour periods twice daily at room temperature. The solutions were agitated on a stir plate to produce subsurface caries-like lesions at constant circulation [15]. The tooth segments were cycled for 28 days for the XPS group.

The pilot samples for PLM evaluation were first pH cycled for 28 days. However, upon sectioning 2 sets of samples, the lesions created were not large enough for quantification. Hence the samples were further cycled for 14 days for a total of 42 days. For PLM, brackets were removed, and additional buccolingual sections 140 to 160 *μ*m in thickness were made from each segment with a Silverstone-Taylor Series 1000 Deluxe hard tissue microtome (Scientific Fabrication, Littleton, CO).

### 2.3. X-Ray Photoelectron Spectrophotometry (XPS)

XPS analysis of the specimens was carried out using a Kratos Axis165 spectrometer (Kratos Analytical Ltd., Manchester, England). Monochromatic Al K-alpha (1486.6 eV) X-rays were used under the following operating conditions: large area slot aperture 700 × 300 *μ*m, charge neutralization filament current of 1.7 A, charge balance of 2.6 V, filament bias 1.3, vacuum readings of 10^−7^ to 10^−8^ torr with the argon gas flow for etching, and the C 1s peak calibrated at 285.0 eV. The scan used covered the 0 to 1,000 eV binding energy range. 

Surface elemental compositions were calculated from the integrated peak areas using Kratos Vision computer programs (V. 2.2.5, Kratos Analytical Ltd., Manchester, England). Calcium 2p (Ca 2p), phosphorus 2s (P 2s), oxygen 1s (O 1s), fluorine 1s (F 1s), silicon 2p (Si 2p), and carbon 1s (C 1s) electron orbits were detected for the binding energy analysis [16]. Four central segments (control group without pH cycling) were analyzed at each of two time points: day 1 (T1) and day 28 (T2). Four lateral tooth segments were included for each of the four test groups (no composite group and three composite groups) and examined at day 1 and day 28. On day 1, the top and bottom exposed enamel of the tooth section was analyzed. After pH cycling, the brackets attached to the composite groups samples were removed on day 28, and the specimens were returned to the XPS instrument for another reading of the top, middle, and bottom sites. Samples were repeated to determine interobserver variability. Altogether for the pilot and experimental XPS trials, 119 sites were tested (Table 1).

### 2.4. Polarized Light Microscopy

An Olympus dual stage polarized light microscope (Model BH-2, Dualmont Corporation, Minneapolis, MN) was used to quantify the demineralized lesions. As shown in Table 1, eight tooth segments were included in each of four test groups (no composite control group and three composite groups). The 140 to 160 *μ*m sections prepared from each tooth segment were wetted with deionized water [17]. Areas of demineralization were centered in the field of view and photographed under maximum illumination at 13.2 times magnification [18]. The lesion depth area for each section was measured using a digital template with a width of 0.5 mm drawn using the Image Pro Plus (Media Cybernetic, Inc., Silver Spring, MD) software program. The buccolingual lesion depths were obtained and then averaged to represent an overall mean lesion depth for each sample.

### 2.5. Bacterial Adhesion Testing

**Figure 2:**
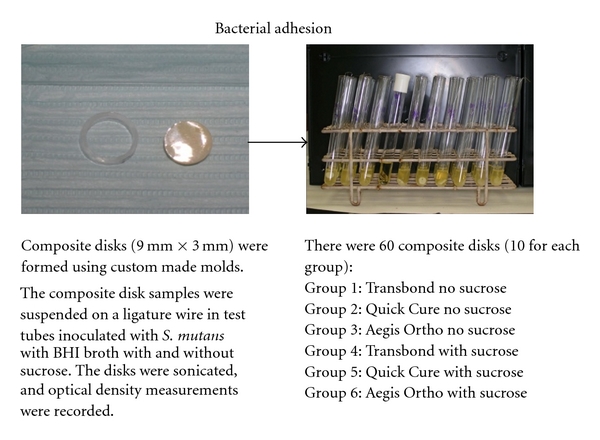
Illustration of bacterial adhesion experiment.

As shown in Figure 2, composite disks (9 mm × 3 mm) from all three adhesives were formed using custom-made molds and light cured for 20 seconds on each side. They were polished with diamond 320 grit paper (Buehler Ltd., Evanston, IL) to remove the oxidative layers and then sonicated to remove debris. Twenty disks of each study composite were made for a total of 60 samples.

The composite disks were sanitized under UV light overnight to remove bacterial contamination [19]. Each group of ten composite samples was suspended on stainless steel orthodontic ligature wires in test tubes containing brain-heart infusion (BHI) broth with or without 1% sucrose [9, 20] and inoculated with *Streptococcus mutans* (strain ATCC 10449) for 48 hours at 37°C. After incubation period, the composite disks were removed and gently dipped 5 times in PBS to dislodge nonadherent bacteria. The disks were then transferred to 3 mL of 1 N NaOH and sonicated for 6 minutes to dislodge the attached *S. mutans*. The absorbance of *S. mutans *suspensions was measured at 550 nm (Spectronic 601, Milton Roy, Rochester, NY). Composite disks in BHI broth alone acted as controls.

### 2.6. Data Analysis

The results were evaluated by SPSS software (SPSS Inc., Chicago, IL). Kruskal-Wallis tests and Mann-Whitney tests were used to analyze the differences in mass concentration of all ions listed above in the XPS methodology paragraph. These tests were also applied in PLM to distinguish differences in lesion size at *P* ≤ 0.05. Results from bacterial adhesion testing with and without sucrose were compared by *t*-test (*P* < 0.01) or one-way ANOVA and Scheffé test (*P* < 0.05). Nonparametric evaluations were used to analyze the subgroups due to small sample sizes.

## 3. Results

### 3.1. XPS Determinations

After examining the three test groups of composite samples under XPS, it was noted that the ACP orthodontic adhesive was not homogeneous. Carbon values did not change in the control group samples that were not pH cycled. Also, there were no significant differences in results of the various analyses between different locations on the tooth surfaces.

**Figure 3:**
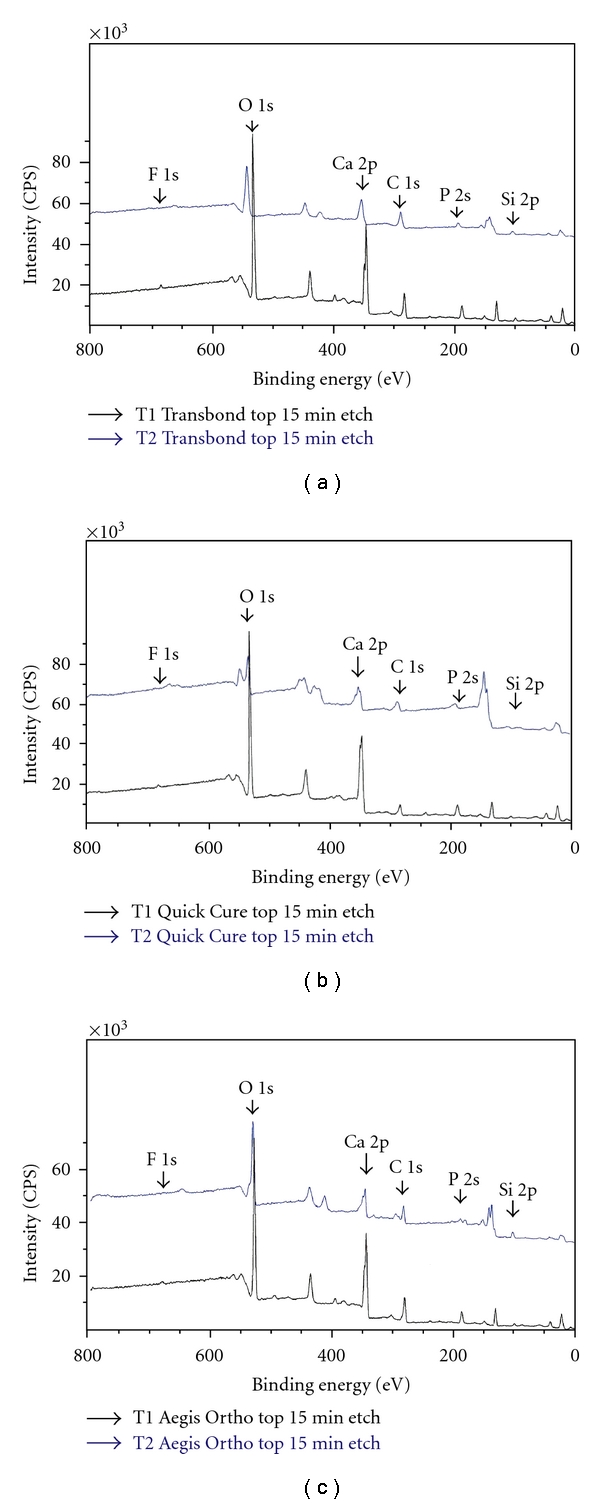
XPS output for the three composite groups (top, Transbond; middle, Quick Cure; bottom, Aegis Ortho) after surface sputter cleaning for 15 minutes to remove atmospheric carbon. Areas under the peaks illustrate the mass concentration percentage present for each element [calcium 2p (Ca 2p), phosphorus 2s (P 2s), oxygen 1s (O 1s), fluorine 1s (F 1s), silicon 2p (Si 2p), and carbon 1s (C 1s) electron orbits]. For Aegis Ortho, carbon and silica increased from T1 to T2 in areas under the peaks and calcium decreased.

At T1, prior to the application of orthodontic adhesives, there were no significant differences (*P* > 0.05) in XPS elemental mass concentrations of Ca, P, O, F. Si, and C in the samples. The Kruskal Wallis Test showed that Transbond XT, Quick Cure, and Aegis Ortho had significant differences in mass elemental concentration; Ca (*P* = 0.032), P (*P* = 0.002), Si (*P* = 0.028), C (*P* = 0.019). In examining the relationship further with Mann-Whitney test, there were no significant differences in mass concentrations between Transbond XT and Quick Cure. However, there were significant differences between Transbond XT and Aegis Ortho; Ca (*P* = 0.007), P (*P* = 0.001), Si (*P* = 0.038), C (*P* = 0.004). Calcium decreased more between the time points in Aegis Ortho (T1, 26.98%; T2, 16.46%) than in Transbond XT (T1, 26.55%; T2, 24.08%). Phosphorus also had a larger decrease while there was a larger increase in silica and carbon in Aegis Ortho than Transbond XT. Aegis Ortho was significantly different from Quick Cure in that the Aegis Ortho samples had less phosphorus (*P* = 0.004) and more surface silica (*P* = 0.013) at T2.  Figure 3 shows representative XPS analyses.

### 3.2. Polarized Light Microscopy

**Figure 4:**
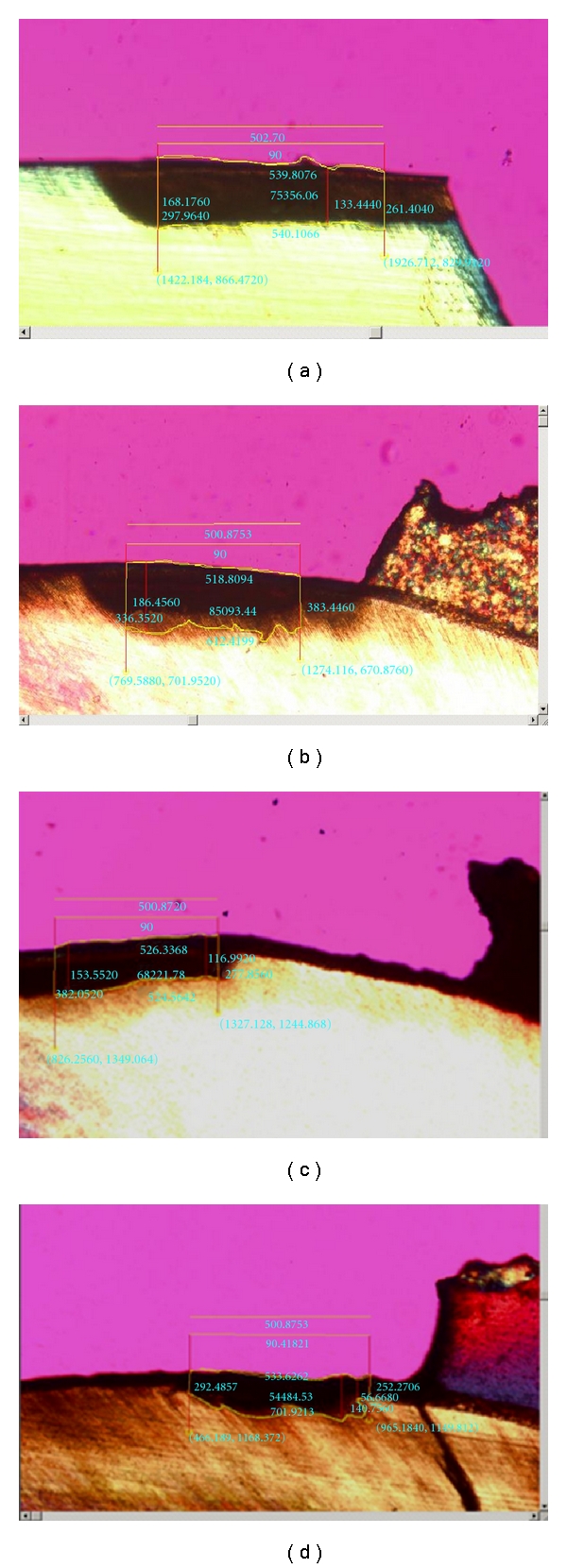
The photomicrographs obtained from polarized light microscopy show representative lesions of each group after 42 days of *in vitro* pH cycling: (a) control group without resin, (b) Transbond XT, (c) Quick Cure, (d) Aegis Ortho. Aegis Ortho had reduction of 20.3% as compared to the no adhesive control enamel group, whereas Transbond XT had an increase in lesion depth area of 3.2% versus the control group.

Twelve sets of lesion images were repeated after one-week interval, and the alpha of reliability coefficient of reliability analysis test was high at 0.994. Lesion depth area and lesion depth results are summarized in Table 2. Kruskal-Wallis testing showed significance differences between the groups (*P* = 0.02). Quick Cure showed a reduction in lesion depth area of 23.6% as compared to control group. Aegis Ortho had reduction of 20.3% versus the control group, whereas Transbond XT had an increase in lesion depth area of 3.2% versus the control group. Representative photomicrographs from polarized light microscopy are shown in Figure 4.

### 3.3. Bacterial Adhesion Testing

**Figure 5:**
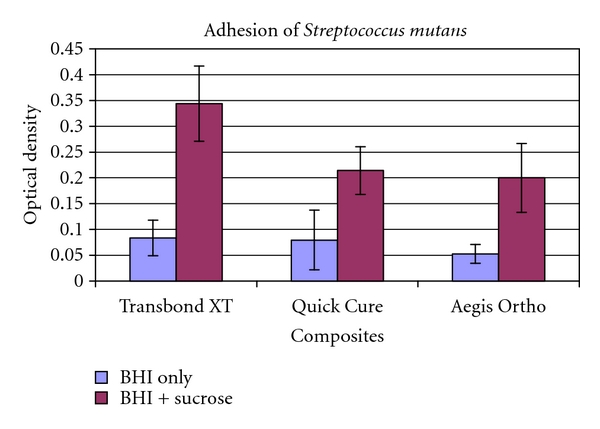
After 48 hours of incubation, Aegis Ortho and Quick Cure exhibited significantly less sucrose-induced *S. mutans* adherence (41.8% reduction in adherence, *P* = 0.004 and 37.7% reduction in adherence, *P* = 0.01 resp.) than Transbond XT without ACP or fluoride (one-way ANOVA, Scheffé test *P* < 0.05).

In the presence of sucrose, *S. mutans* adhered to all three composites (mean OD 0.25 ± 0.11), while no adherence was noted on composites incubated in BHI without sucrose (mean OD 0.07 ± 0.06) (*t*-test, *P* < 0.01). After 48 hours of incubation, Aegis Ortho and Quick Cure exhibited significantly less sucrose-induced *S. mutans* adherence (41.8% reduction in adherence, *P* = 0.004 and 37.7% reduction in adherence, *P* = 0.01, resp.) than Transbond XT without ACP or fluoride (one-way ANOVA, Scheffé test *P* < 0.05) (Figure 5). No significant differences were noted between Quick Cure and Aegis Ortho (*P* > 0.05).

## 4. Discussion

In this *in vitro* study, we examined the alteration of the surface around a fluoride-releasing orthodontic bonding system and an orthodontic adhesive with ACP. The artificial carious lesions created occlusal and gingival to the adhesives were compared, and the amount of bacterial adhesion found on each composite was also investigated. 

Carbon and silica levels increased from T1 to T2 (day 28) since the orthodontic adhesives used to bond the brackets contained a large concentration of carbon and silica. Calcium and phosphorus levels dropped as these ions were dissolved from the teeth samples that were cycled in pH of 4.4. Transbond XT had a smaller amount of decrease in calcium and phosphorus levels than Aegis Ortho. This finding could be due to the release of calcium and phosphorus ions from Aegis Ortho in response to the acid challenge and therefore no longer present on the degraded resin surface when analyzed by XPS. Moreover, the findings are based on a small sample size, and the question of where the released ions go needs to be answered. Richards et al. showed that there was calcium and phosphorus ion release in resin-based calcium phosphate cement and found that the release continued at 56 days [21]. A recent report of Behnan et al. [22] based on a 15-day cycling period showed fewer positive effects of various treatments, possibly because the period was short.

No adhesive control and Transbond XT groups had deeper *in vitro* induced enamel lesions than Quick Cure or Aegis Ortho with ACP. The finding is not surprising because Quick Cure releases ~0.90 *μ*g F^−^/cm^2^/day. Rawls proposed that 0.65–1.30 *μ*g F^−^/cm^2^/day rate is sufficient to inhibit caries initiation in sound enamel near a resin-based dental material [23]. It is well known that fluoride inhibits glycolysis of *S. mutans* and also renders enamel more resistant to acid dissolution [24].

The presence of amorphous calcium phosphate may theoretically shift the equilibrium in favor of strengthening the enamel. However, Aegis Ortho with ACP had more demineralization than Quick Cure in this *in vitro *study. In Derks et al.'s [1] systematic review of *in vivo* studies, caries-inhibiting effect was only considered significant if it was over 50%. They referred to the fluoride-releasing composite tested by Mitchell, Turner, and Trimpeneers and Dermaut which had an overall preventive fraction of 20% and concluded that fluoride-releasing bonding materials have no significant effect in the prevention of demineralization [1].

The addition of sucrose in BHI allowed the formation of sticky glucan polymers by *S. mutans *which resulted in their adherence onto the composite disks. Ahn et al. did not examine sucrose-induced adherence of *S. mutans*, and they observed lower level of bacterial adhesion [25]. In the current study, the fluoride-releasing composite tested had 37.7% less bacterial adhesion than Transbond XT while the ACP composite had 41.8% reduction in *S. mutans* adherence. Badawi et al. did not detect *S. mutans* in the biofilms grown on their fluoride-releasing materials. They concluded that these materials may inhibit bacterial plaque metabolism [26]. There have been only limited investigations regarding the bacterial adhesion of composites containing ACP. The reduction in *S. mutans* adherence may be associated with inhibition of growth or glucosyltransferase that is responsible for adherent glucan synthesis. This needs to be investigated further. The calcium released from the Aegis composite disks was not determined in this study and warrants further investigation.

The mechanical properties of ACP containing orthodontic resin were found to be significantly lower than conventional orthodontic composite [12]. Further research in developing a product that has good mechanical and preventive properties is needed.

## 5. Conclusions

- In this short-term *in vitro* study, incorporation of ACP into orthodontic adhesive material provided reduction in bacterial adhesion and lesion depth formation. The effects were no better than orthodontic adhesive with fluoride, but were better than the resin control.
- These results encourage further investigation in the release rate of ACP, *in vivo* performance, safety/efficacy of composite with ACP and the possibility of its future clinical applications. No study in the literature so far shows complete prevention of enamel decalcification.
- Good oral hygiene and proper diet are still more effective than any adjunctive agent. It is important to continue the development of a preventive agent that can be effectively delivered in the long term in the orthodontic patient who lacks compliance with oral hygiene.

## Figures and Tables

**Table 1:** Details of sample numbers.

XPS samples prepared from 10 premolars	The 119 sites tested were distributed as follows:
	Group 1a (middle tooth sections) controls with no pH cycling (16 sites)*
	Group 1b (middle tooth sections) controls with no composite, pH cycled (16 sites)*
	Group 2 Transbond XT (resin control, pH cycled) (29 sites)**
	Group 3 Quick Cure (resin with fluoride, pH cycled) (29 sites)**
	Group 4 Aegis Ortho (resin with ACP, pH cycled) (29 sites)**
	*4 lateral teeth segments × 2 sites for each tooth (top and
	bottom) × 2 time points [day 1 (T1) and then at day 28 (T2)] = 16 Sites.
	**4 lateral teeth segments × 2 sites at T1 (top and bottom) and
	4 lateral teeth segments × 3 sites at T2 (top, middle under the
	resin, and bottom) + 3 lateral teeth segments × 3 sites repeated
	for interobserver reliability = 29 sites

PLM samples prepared from 30 premolars	For each of the four groups (control, Transbond XT, Quick Cure,
	and Aegis Ortho), there were 8 lateral tooth segments with
	multiple slices prepared for PLM at day 42.

**Table 2:** PLM lesion measurements after 42 days of pH cycling.

Treatment	Lesion area	Average depth	Minimum depth	Maximum depth
Control	7.94 ± 1.14 *μ*m^2^ × 10^4^	155 ± 23 *μ*m	128 ± 22 *μ*m	184 ± 30 *μ*m
Transbond XT	8.19 ± 1.93 *μ*m^2^ × 10^4^	161 ± 38 *μ*m	127 ± 36 *μ*m	193 ± 47 *μ*m
Quick Cure	6.06 ± 1.28 *μ*m^2^ × 10^4^	117 ± 26 *μ*m	85 ± 30 *μ*m	145 ± 28 *μ*m
Aegis Ortho	6.33 ± 1.53 *μ*m^2^ × 10^4^	123 ± 31 *μ*m	79 ± 33 *μ*m	165 ± 27 *μ*m
